# Cross-cultural differences in visual attention: a computational modelling study

**DOI:** 10.1186/1471-2202-16-S1-P204

**Published:** 2015-12-18

**Authors:** Eirini Mavritsaki, Panagiotis Rentzelas

**Affiliations:** 1 Department of Psychology, Birmingham City University, Birmingham, B422SU, UK; 2 Department of Experimental Psychology, University of Oxford, Oxford, OX1 3UD, UK

## 

Literature in visual perception has identified that there are cross-cultural differences in visual perception [1]. Research comparing members of interdepended and collectivist East Asian cultures with independent and individualist European American cultures into picture perception showed that East Asians are more likely to attend the perceptual field as a whole and to focus on context and Westerns to focus on the salient foreground objects [1]. Research on cross-cultural differences has focused on investigating cross-cultural differences related to bottom-up information. Furthermore, research that experimentally manipulated the cultural norms of individualism and collectivism groups managed to attenuate cultural-specific preferences for social factors beneficial in human motivation [2]. Investigating the underlying mechanisms involved in these differences is very important as it can affect everyday tasks, advertisement and many other aspects of our everyday life.

Here we present the first steps of this work, investigating the underlying processes in cross-cultural differences using computational modelling studies. The computational model is based on the spiking Search over Space and Time (sSoTS) model [3], that has been used to simulate Visual Attention task. sSoTS has incorporated mechanisms that allows us to investigate both bottom-up and top-down processes. We show that sSoTS can successfully simulate cross-cultural differences in Visual attention involving bottom-up tasks. Moreover, we expand the studies by making predictions from the computational modelling studies for cross-cultural differences and top-down tasks.
